# Dual-Regularized Feature Selection for Class-Specific and Global Feature Associations

**DOI:** 10.3390/e27020190

**Published:** 2025-02-13

**Authors:** Chenchen Wang, Jun Wang, Yanfei Li, Chengkai Piao, Jinmao Wei

**Affiliations:** 1 College of Computer Science, Nankai University, Tianjin 300350, China; lyfinf@163.com; 2 School of Mathematics and Statistics Science, Ludong University, Yantai 264025, China; junwang@ldu.edu.cn; 3 Information and Navigation College, Air Force Engineering University, Xi’an 710077, China; apark@mail.nankai.edu.cn

**Keywords:** feature selection, feature association, feature manifold

## Abstract

Understanding feature associations is vital for selecting the most informative features. Existing methods primarily focus on global feature associations, which capture overall relationships across all samples. However, they often overlook class-specific feature interactions, which are essential for capturing locality features that may only be significant within certain classes. In this paper, we propose Dual-Regularized Feature Selection (DRFS), which incorporates two feature association regularizers to address both class-specific and global feature relationships. The class-specific regularizer captures the local geometric structure of features within each class. Meanwhile, the global regularizer utilizes a global feature similarity matrix to eliminate redundant features across classes. By combining these regularizers, DRFS selects features that preserve both local interactions within each class and global discriminative power, with each regularizer complementing the other to enhance feature selection. Experimental results on eight public real-world datasets demonstrate that DRFS outperforms existing methods in classification accuracy.

## 1. Introduction

Feature selection (FS) aims to identify the most important features to improve model performance and has been extensively studied [1,2,3]. By removing redundant, noisy, and irrelevant features, FS enhances model performance, reduces computational complexity, and minimizes storage requirements [4,5]. Due to these benefits, FS methods have been successfully applied across a wide range of fields, including bioinformatics [6,7], computer vision [8], multi-view learning [9,10], and recommendation systems [11].

**Figure 1:**
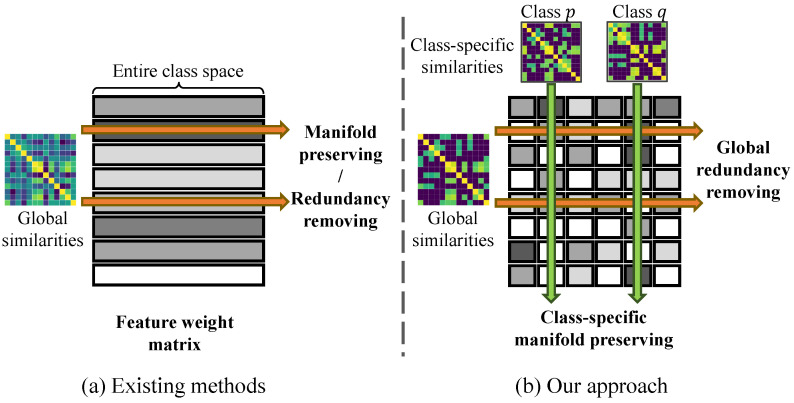
Comparison of global feature association-based methods (**a**) and our approach (**b**). (**a**) Existing methods compute feature associations across the entire dataset to preserve the global feature manifold or eliminate redundant features. (**b**) Our method retains class-specific feature manifolds while removing global feature redundancies.

A critical aspect of FS involves understanding feature associations, which can reveal redundancies or meaningful patterns in data [12,13]. Redundant features, characterized by overlapping information, often degrade model performance [14]. In contrast, meaningful feature associations uncover inherent patterns that are essential for accurate predictions, such as the relationship between temperature and precipitation in weather forecasting. Existing FS methods generally emphasize global feature associations, which capture overall relationships across the entire dataset. These methods generally construct feature similarity matrices and apply regularization techniques to guide the selection process. For example, some approaches utilize cosine similarity matrices to model feature associations and introduce global redundancy regularization to eliminate redundant features [15,16]. Other approaches consider manifold structures in the feature space to select features that preserve these structures [12,17,18]. These methods, as illustrated in Figure 1a, often focus on global interactions across the features, emphasizing paired feature associations or preserving global feature manifolds.

While global feature associations have significantly advanced feature selection, they overlook class-specific interactions that are essential for capturing patterns unique to individual classes. Unlike global associations, which capture relationships across the entire dataset, class-specific associations highlight feature relationships that are meaningful within specific classes but may not generalize to others. Disregarding these localized interactions can result in suboptimal feature selection, as key features relevant to particular classes may be overlooked. The limitations of focusing solely on global associations become apparent in various applications. For example, in medical diagnoses involving multi-class diseases, global associations might highlight features like age and blood pressure as significant across all conditions. However, class-specific interactions, such as the combination of elevated blood sugar and cholesterol levels for diabetes, or the relationship between heart rate variability and blood pressure for cardiovascular diseases, are unique to specific classes. Overlooking such interactions can obscure important class-specific insights, resulting in less effective feature selection.

To address these challenges, we propose Dual-Regularized Feature Selection (DRFS), a novel approach that considers class-specific and global feature associations. By introducing separate regularization terms for global and class-specific associations, our approach aims to enhance the selection of features that are relevant across the entire dataset while capturing unique patterns within individual classes. The class-specific regularizer captures feature interactions unique to each class, while the global regularizer helps reduce redundancy across features by considering the entire dataset. This dual consideration not only improves model performance by selecting more informative features but also enhances interpretability by providing insights into both general and class-specific patterns. As shown in Figure 1b, our method constructs a feature weight matrix that considers class-specific interactions independently while addressing global redundancies. Notably, the class-specific feature similarity matrices exhibit unique structures that differ from one another and from the global matrix, reflecting their localized characteristics. Moreover, the flexibility of DRFS allows it to be integrated with various FS models. In this study, we validate DRFS within a sparse regression framework, which minimizes the difference between selected features and sample labels while incorporating both class-specific and global regularization. Our main contributions are as follows:

- We propose a novel dual-regularization approach for FS, balancing class-specific feature interactions and global redundancy elimination.
- We theoretically show that our dual regularization effectively preserves class-specific feature geometry while addressing global redundancies.
- Extensive experiments on eight real-world datasets show that DRFS consistently outperforms existing FS methods in classification accuracy.

The rest of the paper is organized as follows: Section 2 reviews related work and introduces notation. Section 3 presents the proposed DRFS method. Section 4 details the optimization algorithm, including its complexity and convergence analysis. Section 5 presents the experimental results and analysis. Section 6 concludes the paper.

## 2. Related Works

FS has been studied for many years [1,19,20]. Traditional FS methods are generally categorized into three approaches: filter, wrapper, and embedded methods [1]. Filter methods rely on statistical measures, such as information gain and correlation coefficients, to evaluate and select features [4,5,21]. These methods are among the simplest and fastest techniques for FS. However, they may overlook relationships between features, as they rely solely on individual feature statistics rather than considering feature interactions. Wrapper methods, in contrast, approach FS as a search problem. They evaluate the performance of different feature subsets by training models on each subset, ultimately selecting the optimal one [22,23]. While wrapper methods provide a better understanding of feature relationships compared to filter methods, this advantage comes at a high computational cost, making them less suitable for high-dimensional datasets. Embedded methods integrate feature selection directly into the model training process, selecting the optimal subset while minimizing redundancy and noise [2,13,24]. Many embedded methods employ sparse learning techniques to constrain feature weights during training  [25,26]. These embedded feature selection algorithms have achieved very good performance in many fields [9]. The method proposed in this work belongs to the category of embedded feature selection methods. Below, we provide a brief overview of related works.

Recently, significant attention has been directed towards leveraging feature associations to enhance FS methods. These approaches generally follow two key steps: (1) constructing a feature similarity matrix to capture feature associations, and (2) designing models to select features based on the constructed matrix. For example, Chen et al. introduced the uncorrelated Lasso, which aims to select uncorrelated features by utilizing a squared cosine similarity matrix and $l_{1}$-norm minimization [15]. Building on this, Xu et al. proposed a global redundancy minimization approach (GRMOR), employing orthogonal regression to identify discriminative features [16]. Zhang et al. proposed Interacted Lasso, capturing feature interactions using hypergraphs [27]. Similarly, Cui et al. proposed the structurally interacting elastic net (InElasticNet), which incorporates an information-theoretic criterion for measuring feature associations [28]. They further extended their work to focus on joint feature relevance through the fused Lasso (InFusedLasso) [29]. These methods, predominantly based on Lasso-like frameworks, are effective but limited by their reliance on linear association measures.

Feature manifold learning has recently emerged as a promising direction for feature selection. Xu et al. utilized Pearson correlation coefficients and mutual information to construct feature associations and proposed a redundancy minimization regularization (SFSRM) [26]. Lai et al. improved upon this by using Gaussian functions to refine feature similarity construction and introduced the new regularization term (AGLRM) [30]. Shang et al. explored manifold-preserving methods, assuming that manifold structures exist in the feature space. They proposed dual-graph regularized feature selection (NSSRD) [17] and NNSAFS [18], employing parameter-free and Gaussian-based techniques for feature graph construction. In addition, Roffo et al. proposed infinite feature selection (Inf-FS), which selects features by using the power series of the similarity matrix [31]. They used the Fisher criterion, normalized mutual information, and normalized standard deviation to calculate feature associations. Recently, Cohen et al. proposed a filter FS method called Manifold-based Feature Selection (ManiFeSt), which constructs feature similarity representations for each class and computes feature scores using mean and difference operators [12].

A detailed summary of these methods is presented in Table 1. As shown in Table 1, existing methods mostly consider global feature associations, but not class-specific feature associations. Although ManiFeSt considers class-specific feature associations, it lacks consideration of global feature associations. Our method not only considers global associations, but also class feature associations. Specifically, these methods calculate feature associations in the entire sample space. Additionally, ManiFeSt effectively leverages the feature manifold of each class to improve FS performance. While our method also preserves the structure of individual classes, it differs from ManiFeSt in two key aspects. First, we construct feature similarity matrices using *k*-nearest neighbor graphs, focusing on the most relevant feature interactions. Unlike ManiFeSt, which computes all pairwise feature similarities, our approach better captures the local manifold structure. Second, we incorporate global feature associations to eliminate redundancy, enabling the selection of more discriminative features. These distinctions allow our model to achieve more effective feature selection.

**Notation:** For a matrix $X\in R^{n\times d}$, its (i,j)-th element, *i*-th column, and *j*-th row are denoted as $X_{ij}$, $x_{i}$, and $x^{j}$, respectively. The inverse, transpose, and trace of X are $X^{-1}$, $X^{T}$, and Tr(X), respectively. The $\ell_{2,1}$-norm is ${∥X∥}_{2,1}=\sum_{i}^{n}{∥x^{i}∥}_{2}$. The Frobenius norm of X is given by ${∥X∥}_{F}^{2}=\sum_{i=1}^{d}{∥x_{i}∥}_{2}^{2}$. Let *n*, *d*, and *r* denote the number of samples, features, and classes, respectively. The data matrix is $X\in R^{n\times d}$, where $x^{i}\in R^{1\times d}$ represents the *i*-th sample, and $x_{j}\in R^{n}$ represents the *j*-th feature. The label matrix is $Y\in R^{n\times r}$. The feature similarity matrix for class *l* is $M^{\left(l\right)}\in R^{d\times d}$, and the global feature similarity matrix is $M\in R^{d\times d}$.

## 3. Methods

**Figure 2:**
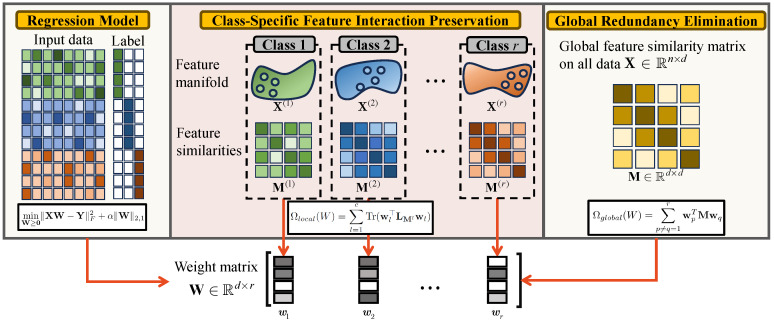
An overview of the proposed DRFS method.

We propose the novel Dual-Regularized Feature Selection (DRFS) model with two complementary regularizations: class-specific feature interaction preservation and global redundancy elimination. The first regularization ensures the preservation of local feature interactions within each class, capturing the underlying feature manifold structure. The second regularization addresses global feature associations, aiming to eliminate redundant features. These complementary strategies combine to select features that retain local geometric relationships and enhance global discriminative power, as shown in Figure 2. To validate the effectiveness of the proposed regularizations, we use a sparse regression model as the baseline. Notably, our regularization technique is designed to function as an adaptable plugin, compatible with various FS models.

### 3.1. Class-Specific Feature Interaction Preservation

Class-specific feature interaction preservation regularization is developed in two stages. First, we construct class-specific feature similarity matrices to capture the local manifold structure. Next, we introduce a regularization strategy based on these matrices to preserve the local feature geometry.

In the first stage, we model the geometric structures of features for each class. Given a dataset X with *r* classes, we partition the data by class labels, such that $X=\{X^{\left(1\right)},X^{\left(2\right)},\ldots ,X^{\left(r\right)}\}$, where $X^{\left(l\right)}\in R^{n^{\left(l\right)}\times d}$ represents the sample matrix for class *l* with $n^{\left(l\right)}$ samples. $\sum_{i=1}^{l}n^{\left(l\right)}=n$ is the total number of samples. For each class *l*, we define a feature similarity matrix $M^{\left(l\right)}\in R^{d\times d}$ that quantifies the relationships between features. To capture non-linear interactions between features, we use the Radial Basis Function (RBF) kernel, which is commonly employed for manifold learning [12]. To model local feature geometric structure, we define the feature similarity matrix for class *l* as:

(1) $$\;M_{ij}^{\left(l\right)}=\left\{\begin{array}{cc} e^{-∥x_{i}^{\left(l\right)}-x_{j}^{\left(l\right)}{∥}_{2}^{2}/\sigma_{l}^{2}}, & x_{i}^{\left(l\right)}\in N_{k}\left(x_{j}^{\left(l\right)}\right)\;\mathrm{or}\;x_{j}^{\left(l\right)}\in N_{k}\left(x_{i}^{\left(l\right)}\right)\\ 0, & \mathrm{otherwise}, \end{array}\right.$$

where $x_{i}^{\left(l\right)}$ and $x_{j}^{\left(l\right)}$ represent the *i*-th and *j*-th features of class *l*, and $N_{k}\left(x_{i}^{\left(l\right)}\right)$ is the set of *k*-nearest neighbors of feature $x_{i}^{\left(l\right)}$. $M_{ij}^{\left(l\right)}$ reflects the relationship between features *i* and *j* within class *l*. The *k*-nearest neighbors approach captures the most relevant features, providing a more accurate representation of the feature manifold compared to pairwise similarity, as shown in [12]. Moreover, the storage complexity of our approach is O(dk), which is more efficient than the $O(d^{2})$ required by pairwise methods.

In the second stage, we apply the feature similarity matrix to solve the following optimization problem, which helps preserve the local feature interactions:

(2) $$\min_{w_{l}\geq 0}\mathrm{Tr}\left(w_{l}^{⊤}L_{M^{\left(l\right)}}w_{l}\right)=\sum_{i,j}^{d}{\left(W_{il}-W_{jl}\right)}^{2}M_{ij}^{\left(l\right)},$$

where $L_{M^{\left(l\right)}}=D^{\left(l\right)}-M^{\left(l\right)}$ is the Laplacian matrix of $M^{\left(l\right)}$, and $D^{\left(l\right)}$ is the degree matrix with entries $D_{ii}^{\left(l\right)}=\sum_{j=1}^{d}M_{ij}^{\left(l\right)}$. The vector $w_{l}$ represents the feature weights for class *l*. If $M_{ij}^{\left(l\right)}$ is large, indicating that features *i* and *j* are similar in class *l*, their contributions and corresponding weights $w_{il}$ and $w_{jl}$ should be similar, ensuring that highly correlated interactive features within a specific class are selected simultaneously.

For all *r* classes, we aggregate the class-specific regularization terms as follows:

(3) $$\min_{W\geq 0}\;\Omega_{local}\left(W\right)=\sum_{l=1}^{r}\mathrm{Tr}\left(w_{l}^{⊤}L_{M^{\left(l\right)}}w_{l}\right).$$

where $W=[w_{1},\cdots ,w_{r}]$ is the feature weight matrix, which is solved in a column-wise fashion.

### 3.2. Global Feature Redundancy Elimination

While class-specific regularization captures interactions within classes, highly correlated features may generate redundancy. To address this, we propose a global feature redundancy elimination regularization that removes redundancy across classes. First, we construct a global feature similarity matrix $M\in R^{d\times d}$ to represent feature associations across the entire feature space. The global feature similarity matrix is defined as

(4) $$M_{ij}=\left\{\begin{array}{cc} e^{-∥x_{i}-x_{j}{∥}_{2}^{2}/\sigma^{2}}, & x_{i}\in N_{k}\left(x_{j}\right)\;\mathrm{or}\;x_{j}\in N_{k}\left(x_{i}\right)\\ 0, & \mathrm{otherwise}, \end{array}\right.$$

where $N_{k}\left(x_{i}\right)$ denotes the set of *k*-nearest neighbors of feature $x_{i}$. This ensures that only the most correlated feature associations are captured. In contrast to the local feature interactions within a specific class, as measured in (1), the global matrix in (4) quantifies feature redundancy across all classes.

Interestingly, consider two redundancy features *i* and *j*, which exhibit consistently high similarity across all classes. In other words, for every class *l*, the condition $x_{i}^{\left(l\right)}\in N_{k}\left(x_{j}^{\left(l\right)}\right)\;\mathrm{or}\;x_{j}^{\left(l\right)}\in N_{k}\left(x_{i}^{\left(l\right)}\right)$ holds. Consequently, the global condition $x_{i}\in N_{k}\left(x_{j}\right)\;\mathrm{or}\;x_{j}\in N_{k}\left(x_{i}\right)$ is also satisfied. We now establish the connection between the global feature similarity in (4) and class-specific similarities in (1) in the following theorem.

**Theorem 1.**  *Let $\sigma =\sigma_{1}=\cdots =\sigma_{r}$ and assume that features i and j exhibit redundancy in each class l. Then, the global similarity between features can be expressed as*

(5) $$M_{ij}=M_{ij}^{\left(1\right)}M_{ij}^{\left(2\right)}\cdots M_{ij}^{\left(r\right)}.$$

**Proof.**  By exploiting the decomposition of the Euclidean distance across classes, we have

(6) $$M_{ij}=e^{-∥x_{i}-x_{j}{∥}_{2}^{2}/\sigma^{2}}=e^{-\sum_{l}^{r}{∥x_{i}^{\left(l\right)}-x_{j}^{\left(l\right)}∥}_{2}^{2}/\sigma^{2}}=\Pi_{l}^{r}e^{-∥x_{i}^{\left(l\right)}-x_{j}^{\left(l\right)}{∥}_{2}^{2}/\sigma^{2}}=\Pi_{l}^{r}M_{ij}^{\left(l\right)}$$

This completes the proof.    □

From Theorem 1, the global feature similarity matrix aggregates local feature associations across all classes, assigning high values to redundant features. In practice, the assumptions in Theorem 1 are challenging to satisfy due to the computation of feature similarity matrices using *k*-nearest neighbors with varying $\sigma_{l}$. Nonetheless, the aggregation approach described in Theorem 1 still ensures that redundant feature associations are preserved in M, either across all classes or within specific classes.

To eliminate global redundancy, we introduce the following regularization term:

(7) $$\min_{W\geq 0}\Omega_{global}\left(W\right)=\sum_{p=1}^{r}\sum_{q\neq p}^{r}w_{p}^{T}{\mathrm{Mw}}_{q},$$

where $w_{p}$ and $w_{q}$ represent the *p* and *q* columns of W, respectively. Minimizing (7) reduces redundancy by encouraging the model to eliminate features that exhibit high correlation across different classes. To better understand the effect of minimizing $\sum_{p=1}^{r}\sum_{q\neq p}^{r}w_{p}^{T}{\mathrm{Mw}}_{q}$, we expand (7) as follows:

(8) $$\begin{array}{cc} \; & \sum_{p=1}^{r}\sum_{q\neq p}^{r}w_{p}^{T}{\mathrm{Mw}}_{q}=\sum_{p=1}^{r}\sum_{q\neq p}^{r}\left(\sum_{i}^{d}\sum_{j}^{d}W_{ip}W_{jq}M_{ij}\right)\\ = & \sum_{i,j}^{d}\left(\sum_{p=1}^{r}\sum_{q\neq p}^{r}W_{ip}W_{jq}M_{ij}\right)=\sum_{i,j}^{d}\left(\sum_{p=1}^{r}W_{ip}\left(\sum_{q\neq p}^{r}W_{jq}\right)M_{ij}\right)\\ = & \sum_{i,j}^{d}\left(\right|w^{i}\left|\right|w^{j}\left|M_{ij}-\sum_{p=1}^{r}W_{ip}W_{jp}M_{ij}\right)\\ = & \sum_{i,j}^{d}\left(\right|w^{i}\left|\right|w^{j}\left|M_{ij}\right)+\sum_{i,j}^{d}\left(\sum_{p=1}^{r}W_{ip}W_{jp}\left(-M_{ij}\right)\right), \end{array}$$

where $|w^{i}|=\sum_{p=1}^{r}W_{ip}$ represents the weight accumulation of w, which indicates the importance of feature *i*. Minimizing the formula above is equivalent to minimizing the two components separately. We analyze each term as follows. (1) For the first term: If $M_{ij}$ is large, it indicates that features *i* and *j* are highly correlated. In this case, if $|w^{i}\left|>\right|w^{j}|$, the weight $|w^{j}|$ will decrease to minimize this term. When features *i* and *j* are redundant, the model reduces the weight of one feature to eliminate redundancy. (2) For the second term: If $-M_{ij}$ is large (i.e., $M_{ij}$ is small), it indicates that the features *i* and *j* are less correlated. To minimize this term, the weights $w_{ip}$ and $w_{jp}$ for class *p* will differ, emphasizing the distinction between two features. When the features are not redundant, they should contribute to different classes to enhance the model’s discriminative ability. These observations highlight the significance of our approach. It clearly demonstrates that (7) effectively removes redundant features between classes and within classes.

### 3.3. Unified Objective Function

We introduce the dual-regularization term in (9), which consists of the class-specific regularization term, $\Omega_{local}\left(W\right)$, and the global regularization term, $\Omega_{global}\left(W\right)$:

(9) $$\begin{array}{cc} R(W) & =\min_{W\geq 0}\frac{1}{r}\Omega_{local}\left(W\right)+\frac{1}{r(r-1)}\Omega_{global}\left(W\right)\\ \; & =\frac{1}{r}\sum_{l}Tr\left(w_{l}^{T}L_{M^{\left(l\right)}}w_{l}\right)+\frac{1}{r(r-1)}\sum_{p}\sum_{q\neq p}^{r}w_{p}^{T}{\mathrm{Mw}}_{q}. \end{array}$$

Here, $\frac{1}{r}$ and $\frac{1}{r(r-1)}$ are normalization factors that balance the contributions of the class-specific and global regularization terms. The dual-regularization term, R(W), effectively preserves the local feature structures while simultaneously reducing global feature redundancy. This regularization term can be easily integrated into existing FS models, enriching them with additional feature association information. In this work, we apply it to the following sparse regression model, a simple yet widely used approach. The final objective function is defined as

(10) $$\min_{W\geq 0}{∥\mathrm{XW}-Y∥}_{F}^{2}+\alpha {∥W∥}_{2,1}+\beta \left(\frac{1}{r}\Omega_{local}\left(W\right)+\frac{1}{r(r-1)}\Omega_{global}\left(W\right)\right).$$

where Y is the label matrix and ${∥W∥}_{2,1}$ is the sparse norm used to enforce sparsity in W. The constraint W≥0 facilitates the selection of features with meaningful physical interpretations [24]. The parameter α controls the sparsity level, and β determines the strength of the dual-regularization term. Note that we use β to scale both the class-specific and global regularization terms. The normalization factors $\frac{1}{r}$ and $\frac{1}{r(r-1)}$ ensure these terms have comparable magnitudes, thus simplifying the model by reducing the number of parameters.

## 4. Optimization

In this section, we propose an efficient algorithm to solve the objective function in (10), leveraging the Augmented Lagrangian Multiplier (ALM) method [32]. First, we introduce a slack variable Z and reformulate (10) as follows:

(11) $$\begin{array}{c} \min_{W}{∥\mathrm{XW}-Y∥}_{F}^{2}+\alpha {∥W∥}_{2,1}+\beta \left(\frac{1}{r}\Omega_{local}\left(Z\right)+\frac{1}{r(r-1)}\Omega_{global}\left(Z\right)\right)\\ \;\;\;s.t.W\geq 0,Z=W,Z\geq 0. \end{array}$$

The corresponding augmented Lagrangian function is

(12) $$\begin{array}{c} \min_{W\geq 0,Z\geq 0}{∥\mathrm{XW}-Y∥}_{F}^{2}+{\alpha ∥W∥}_{2,1}+\mu {∥W-Z+\frac{\Lambda }{\mu }∥}_{F}^{2}\\ +\beta (\frac{1}{r}\Omega_{local}\left(Z\right)+\frac{1}{r(r-1)}\Omega_{global}\left(Z\right)), \end{array}$$

where Λ is the Lagrange multiplier and μ is the penalty parameter. We develop an alternatively iterative algorithm to solve it.

### 4.1. Optimize W

When Z is fixed, problem (12) can be transformed to

(13) $$\begin{array}{c} \min_{W\geq 0}{∥\mathrm{XW}-Y∥}_{F}^{2}+\mu ∥W-Z+\frac{\Lambda }{\mu }{∥}_{F}^{2}+\alpha {∥W∥}_{2,1}. \end{array}$$

The corresponding Lagrangian function is

(14) $$\begin{array}{c} \min_{W}{∥\mathrm{XW}-Y∥}_{F}^{2}+\mu {∥W-Z+\frac{\Lambda }{\mu }∥}_{F}^{2}+\alpha Tr\left(W^{T}\mathrm{DW}\right)+Tr\left(\Sigma W\right), \end{array}$$

where Σ is Lagrange multipliers for the constrains W≥0, and D is the diagonal matrix with $D_{ii}=1/2{∥W^{i}∥}_{2}$. Setting the derivative with respect to W to zero, we have

(15) $$\left(X^{T}X+\mu I+\alpha D\right)W-X^{T}Y-\mu Z+\Lambda +\Sigma =0.$$

Using the Karush–Kuhn–Tucker condition $\Sigma_{ij}W_{ij}$, we have

(16) $${\left(\left(X^{T}X+\mu I+\alpha D\right)W\right)}_{ij}W_{ij}={\left(X^{T}Y+\mu Z-\Lambda \right)}_{ij}W_{ij}$$

To solve this, we decompose any matrix O into two non-negative parts, $O=O^{+}-O^{-}$. Then, the update rule for W is

(17) $$W_{ij}\leftarrow W_{ij}\frac{{\left({\left(X^{T}X+\mu I+\alpha D\right)}^{-}W+{\left(X^{T}Y+\mu Z-\Lambda \right)}^{+}\right)}_{ij}}{{\left({\left(X^{T}X+\mu I+\alpha D\right)}^{+}W+{\left(X^{T}Y+\mu Z-\Lambda \right)}^{-}\right)}_{ij}}$$

### 4.2. Optimize Z

With fixed W, problem (12) becomes

(18) $$\begin{array}{c} \min_{Z}\beta \left(\frac{1}{r}\Omega_{local}\left(Z\right)+\frac{1}{r(r-1)}\Omega_{global}\left(Z\right)\right)+\mu {∥W-Z+\frac{\Lambda }{\mu }∥}_{F}^{2}. \end{array}$$

The above formula can be reformulated as

(19) $$\begin{array}{c} \min_{z_{l}}\frac{\beta }{r}\sum_{l}Tr\left(z_{l}^{T}L_{M^{\left(l\right)}}z_{l}\right)+\frac{\beta }{r(r-1)}\sum_{l}^{r}\sum_{q\neq l}^{r}Z_{l}^{T}{\mathrm{Mz}}_{q}+\mu \sum_{l}{∥w_{l}-z_{l}+\frac{\Lambda_{l}}{\mu }∥}_{2}^{2}. \end{array}$$

Taking the derivative with respect to $z_{l}$ and setting it to zero, we have

(20) $$2\frac{\beta }{r}L_{M^{\left(l\right)}}z_{l}+2\frac{\beta }{r(r-1)}\sum_{q\neq l}^{r}{\mathrm{Mz}}_{q}+2\mu \left(z_{l}-w_{l}-\frac{\Lambda_{l}}{\mu }\right)=0$$

Then, we have

(21) $$\left(\frac{\beta }{r}L_{M^{\left(l\right)}}+\mu I\right)z_{l}=\mu w_{l}+\Lambda_{l}-\frac{\beta }{r(r-1)}\sum_{q\neq l}^{r}{\mathrm{Mz}}_{q}$$

The solution of $z_{l}$ is

(22) $$z_{l}={\left(\frac{\beta }{r}L_{M^{\left(l\right)}}+\mu I\right)}^{-1}\left(\mu w_{l}+\Lambda_{l}-\frac{\beta }{r(r-1)}\sum_{q\neq l}^{r}{\mathrm{Mz}}_{q}\right)$$

To guarantee that the solution Z remains within the feasible region (Z≥0), an element-wise projection is applied:

(23) $$z_{l}=\max\left(z_{l},0\right)$$

where the max(·) operation is applied element-wise, projecting $z_{l}$ into the feasible region.

The detailed steps for DRFS are outlined in Algorithm 1.
**Algorithm 1** DRFS**Input**: Data matrix X, label matrix Y, α, β.Initialize $\mu =1,\;\rho =1.1,\;\mu_{max}=10^{8}$.**Output**: Feature weight matrix W.  1:Compute feature similarity matrices $M^{\left(1\right)},\;\ldots ,\;M^{\left(r\right)}$, M  2:**while** not converaged **do**  3:   Update W by $\;\;W_{ij}\leftarrow W_{ij}\frac{{\left({\left(X^{T}X+\mu I+\lambda D\right)}^{-}W+{\left(X^{T}Y-\mu Z+\Lambda \right)}^{+}\right)}_{ij}}{{\left({\left(X^{T}X+\mu I+\lambda D\right)}^{+}W+{\left(X^{T}Y-\mu Z+\Lambda \right)}^{-}\right)}_{ij}}$;  4:   Compute diagonal matrix D as $D_{ii}=1/2{∥W^{i}∥}_{2}$;  5:   **for** l=1 **to** *r* **do**  6:       Update $z_{l}$ by $z_{l}={\left(\frac{1}{r}L_{M^{\left(l\right)}}+\mu I\right)}^{-1}\left(\mu w_{l}+\Lambda_{l}-\frac{1}{r(r-1)}\sum_{k\neq l}^{r}{\mathrm{Mz}}_{k}\right)$;  7:       $z_{l}=\max\left(z_{l},0\right)$;  8:   **end for**  9:   Update Λ←Λ+μ(W−Z);10:   Update $\mu =\min(\rho \mu ,\mu_{max})$;11:**end while**

### 4.3. Complexity and Convergence

DRFS is solved using Algorithm 1, which primarily involves optimizing W and Z. The computational complexity of optimizing W is $O(d^{2}n)$, while the most computationally expensive step is the optimization of $z_{l}$, requiring matrix inversion with a complexity of $O(d^{3})$. Thus, the overall time complexity is $O(d^{2}n+d^{3}r)$. In practice, the number of classes *r* is much smaller than both the number of samples *n* and the number of features *d* (i.e., r≪d,r≪n). As a result, the computational overhead introduced by *r* is relatively small.

Problems (13) and (18) are convex, ensuring reliable optimization. The convergence of W, updated via (17), has been theoretically established in [24]. The convergence of D is supported by the analysis in [25]. Additionally, the variable Z has a closed-form solution, ensuring its optimality in each iteration. However, it is difficult to prove the overall convergence due to the complexity of the ALM method. Fortunately, we have empirically validated the convergence of our method, as demonstrated in Section 5.6.

## 5. Experiments

In this section, we design four experiments to evaluate the effectiveness of DRFS, focusing on the following aspects: (1) The performance of DRFS with SVM and 1NN classifiers. (2) Ablation studies to validate the role of class-specific and global regularization terms. (3) Sensitivity analysis to investigate the impact of the parameters α and β, as well as the sensitivity to class-specific and global regularization terms. (4) A convergence analysis of Algorithm 1.

### 5.1. Datasets

We conduct experiments on eight public benchmark datasets, including four bioinformatics datasets (lung_discrete [33], SRBCT [34], LUNG [35], GLIOMA), one spoken letter recognition dataset (Isolet [36]), and three image datasets (mfeat [37], Yale [38], warpPIE10P [38]). The details of all the datasets are summarized in Table 2. The lung_discrete dataset contains 73 samples described by 325 gene expressions. The Isolet dataset consists of 1560 spoken letter samples, each represented by 617 features. The mfeat dataset consists of 2000 samples of handwritten digits (0–9), characterized by 649 features. The Yale dataset includes 165 facial images of size 32 × 32, with each pixel treated as a feature. The warpPIE10P dataset comprises 210 images of 10 individuals, each represented by 2420 features (44 × 55). The SRBCT dataset consists of 83 samples, each described by 2308 gene expression features. The LUNG dataset includes 203 samples, each characterized by 3312 gene expression features. The GLIOMA dataset contains 50 samples, with each represented by 4434 gene expression features. The datasets used for the experiments are derived from the scikit-feature selection repository (https://jundongl.github.io/scikit-feature/datasets.html, accessed on 13 January 2025) and the UCI machine learning repository (https://archive.ics.uci.edu/ml/datasets, accessed on 13 January 2025).

### 5.2. Experiment Settings

We evaluate the performance of DRFS by comparing it with several state-of-the-art feature selection methods. Specifically, we include three traditional filter-based methods: Fisher Score [5], Gini Index [21], and RelieF [4]. Additionally, we compare DRFS with RJFWL [24], a non-negative constrained sparse regression method. Most importantly, we compare DRFS with three feature graph-based methods: GRMOR [16], Inf-FS [31], and ManiFeSt [12]. GRMOR [16] computes feature similarity using squared cosine similarities to identify and eliminate redundant features. Inf-FS [31] adopts a weighted feature similarity calculation approach and leverages the power series of matrices or Markov chains to determine feature scores. ManiFeSt [12] calculates feature similarity matrices under different classes and calculates feature scores based on manifold learning. The code for Fisher, Gini Index, and RelieF is available at https://github.com/jundongl/scikit-feature, accessed on 13 January 2025. The code for Inf-FS can be found at https://github.com/giorgioroffo/Infinite-Feature-Selection, accessed on 13 January 2025. The code for ManiFeSt is available at https://github.com/DavidCohen2/ManiFeSt, acessed on 12 February 2025. Our code is available at https://github.com/Wangchenchen233/DRFS, accessed on 13 January 2025.

We evaluate the performance of feature selection using classification accuracy on the selected features as the evaluation metric. We employ 5-fold cross-validation to assess performance with linear SVM and 1-Nearest Neighbor (1-NN) as classifiers. The parameter *C* for the linear SVM is fixed at 1 across all experiments, as in [39]. For all datasets, the range of selected features is set to {10,20,30,40,50,60,70,80,90,100}. For ManiFeSt, we use a naive one-vs-one extension for the multi-class setting. For global feature similarity matrix, we set $\sigma^{2}=mean(∥x_{i}-x_{j}{∥}_{2}^{2}),\;i,\;j=1,\;\cdots ,\;d$. Similarly, for class-specific feature similarities, we set $\sigma_{l}^{2}=mean(∥x_{i}^{\left(l\right)}-x_{j}^{\left(l\right)}{∥}_{2}^{2}),\;i,\;j=1,\;\cdots ,\;d$. The number of feature neighbors *k* is set to 10. For DRFS, we set the parameter search interval to {0.001,0.01,0.1,1,10,100,1000}. For RJFWL and GRMOR, the parameters are adjusted within the interval {0.001,0.01,0.1,1,10,100,1000}. For Inf-FS, we adjust the parameters at {0.2,0.4,0.6,0.8}. All experiments are conducted on a PC with an Intel(R) Core(TM) i9-7900X CPU @ 3.3 GHz and 64 GB RAM.

### 5.3. Results and Analysis

**Figure 3:**
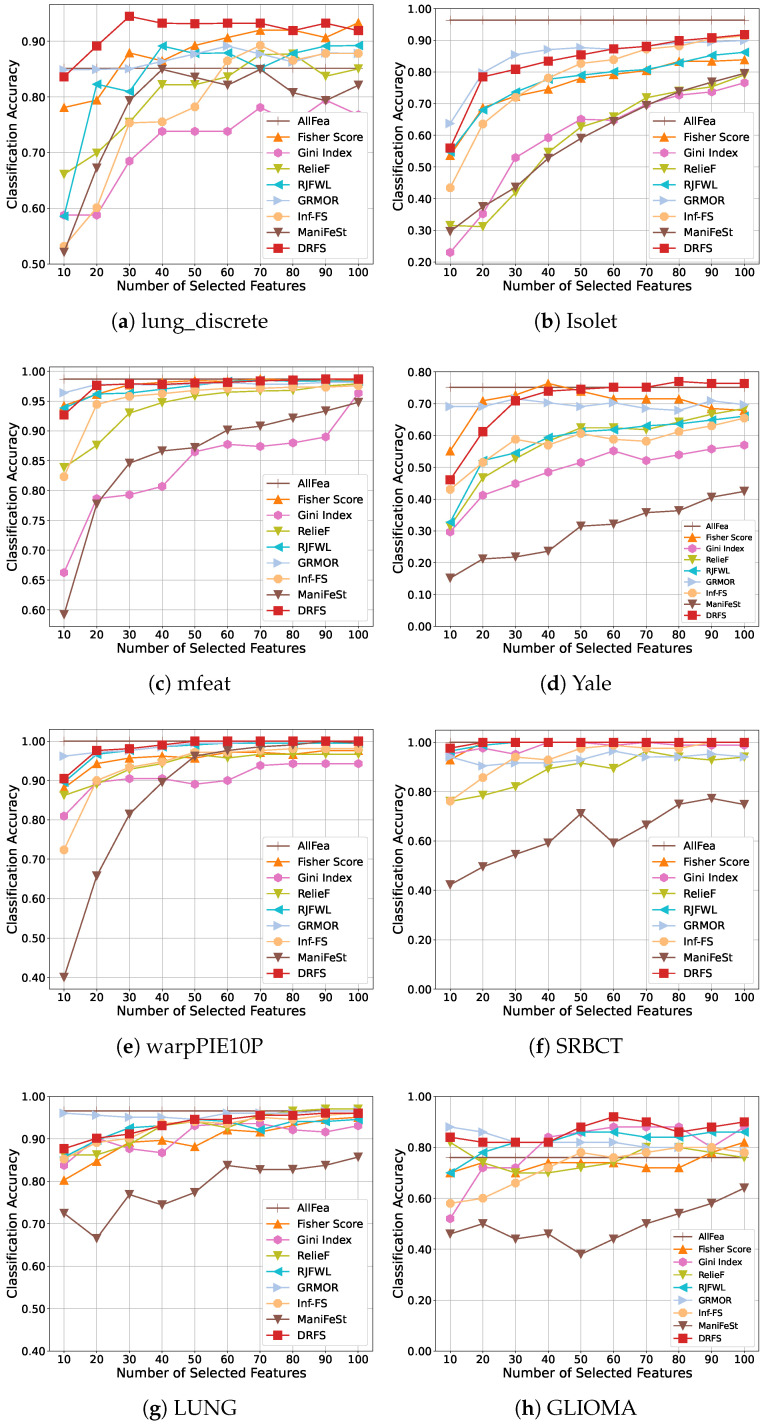
Classification accuracy of the SVM classifier with different number of selected features on eight datasets.

**Figure 4:**
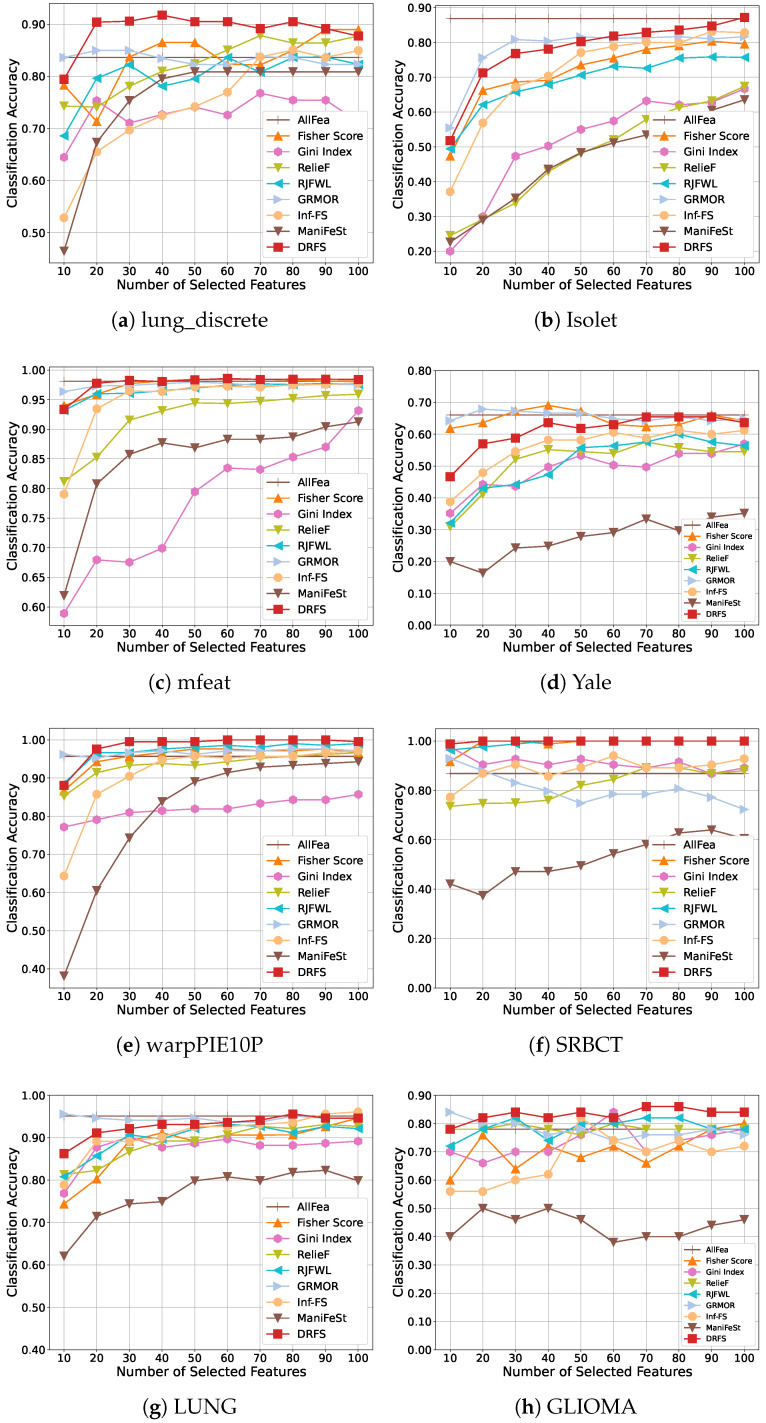
Classification accuracy of the 1-NN classifier with different number of selected features on eight datasets.

Figure 3 and Figure 4 illustrate the classification accuracy results achieved by the SVM and 1-NN classifiers, respectively. Table 3 and Table 4 summarize the best performance achieved by each method. Overall, DRFS consistently outperforms competing approaches across multiple datasets and classifiers. When compared with traditional filter-based methods such as Fisher Score, Gini Index, and RelieF, DRFS demonstrates superior classification accuracy. By incorporating feature association regularization, DRFS also outperforms the sparse regression-based method RJFWL, showcasing its ability to effectively capture feature interactions.

Compared to other feature association methods, DRFS achieves the best overall performance. Notably, DRFS outperforms GRMOR, which focuses solely on global feature redundancy minimization. For instance, on the lung_discrete and Yale datasets, DRFS improves classification accuracy by 5.3% and 5.5% with the SVM classifier, respectively. Furthermore, DRFS outperforms other advanced graph-based and manifold learning methods, including Inf-FS and ManiFeSt. For instance, as shown in Table 4, DRFS achieves higher classification accuracy than ManiFeSt on all datasets with the 1-NN classifier, outperforming Inf-FS on all datasets except for LUNG. This result underscores DRFS’s ability to leverage both global and local feature associations effectively, resulting in improved feature selection and classification outcomes.

DRFS achieves the best performance on biological datasets, such as the lung_discrete and GLIOMA datasets, as shown in Figure 3 and Figure 4. Compared to the optimal results in Table 3, DRFS achieves a 4% improvement on the GLIOMA dataset. Similarly, compared to Table 4, DRFS improves by 2.76% on the lung_discrete dataset. On balanced datasets such as Isolet, mfeat, Yale, and warpPIE10P, DRFS delivers the best results when using the SVM classifier. For unbalanced datasets, including lung_discrete, SRBCT, and GLIOMA, DRFS consistently outperforms all other methods. However, on the particularly imbalanced LUNG dataset, DRFS achieves only the second-best performance, highlighting a potential limitation in handling extreme data imbalance.

**Figure 5:**
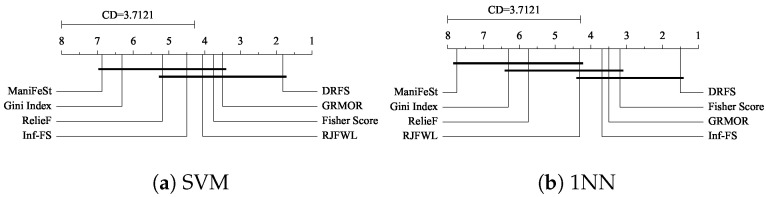
Critical difference diagram by the Bonferroni–Dunn post hoc test (significance level of 0.05).

To statistically compare the performance of the methods, we apply the Friedman test, followed by the Bonferroni–Dunn post hoc analysis. The results of the Friedman test, presented in Table 5, reject the null hypothesis—that all methods perform equivalently—at a significance level of 0.05. Figure 5 displays the critical distance (CD) diagrams, with a critical distance of 3.7121, which indicates that the methods are statistically similar within this threshold. It is noteworthy that DRFS consistently ranks first when evaluated using both the SVM and 1NN classifiers, underscoring its superior performance across these classifiers.

### 5.4. Ablation Study

Table 6 and Table 7 evaluate the impact of the two regularization terms in DRFS on classification performance. These regularization terms are class-specific regularization and global regularization, which together form four different module configurations. In Table 6 and Table 7, the column labeled “Modules” indicates the specific combinations of these regularization terms used in each model. A check mark (√) signifies that the corresponding regularization term has been selected for that model configuration.

The results demonstrate that the combination of both regularization terms consistently outperforms configurations where only one term is used or no regularization is applied. Using a single regularization term results in notable performance gains compared to the absence of regularization, indicating that even partial regularization contributes to effective feature selection. For instance, combining both terms results in relative improvements of 8.5% and 12.9% on the lung_discrete and Yale datasets with SVM, respectively, compared to no regularization. Similarly, with the 1-NN classifier, combining both regularization terms achieves improvements of 9.2% and 10.6% on the same datasets. Interestingly, on the mfeat dataset, the class-specific regularization term alone achieves performance comparable to the combined approach. This observation underscores the critical role of capturing class-specific feature associations, a key contribution of DRFS. These findings highlight the importance of incorporating both global and class-specific regularization terms to enhance classification accuracy.

### 5.5. Parameter Sensitivity

**Figure 6:**
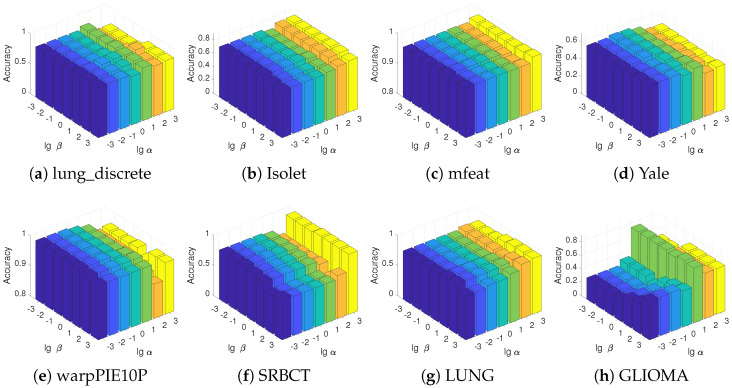
Parameter sensitivity study of DRFS with respect to α and β across all datasets. The parameters α and β correspond to the sparse norm and dual regularizations, respectively.

DRFS introduces two key parameters, α and β, that significantly influence its performance. The parameter α impacts performance by controlling the sparsity of the feature weight matrix. The parameter β governs the strength of the feature association regularization, influencing the model’s ability to capture feature interactions. To evaluate their sensitivity, we fix the number of selected features to 50 and examine their impact on classification accuracy. The results are illustrated in Figure 6. It is evident that optimal performance arises from a balanced interplay between α and β. Notably, the parameter α exerts a more pronounced effect on model performance. When α is set too high, the feature selection matrix becomes overly sparse, leading to the selection of only a few features. Consequently, the model may lack sufficient information for effective learning. Conversely, when α is too low, the model selects many features, including redundant or irrelevant ones, which degrades performance. In comparison, the model’s performance shows weaker sensitivity to variations in β. This relative robustness can be attributed to the fact that β regulates feature association regularization, which refines feature relationships rather than directly influencing sparsity. These findings highlight the importance of careful parameter tuning in DRFS.

DRFS regulates the strength of class-specific and global regularizations through the normalization coefficients $\frac{1}{r}$ and $\frac{1}{r(r-1)}$, respectively. To further investigate the effect of these regularization terms on model performance, we introduce two additional regularization parameters, $\lambda_{1}$ and $\lambda_{2}$, as represented in the following optimization formulation:

(24) $$\begin{array}{c} \min_{W\geq 0}{∥\mathrm{XW}-Y∥}_{F}^{2}+\alpha {∥W∥}_{2,1}+\frac{\lambda_{1}}{r}\Omega_{local}\left(W\right)+\frac{\lambda_{2}}{r(r-1)}\Omega_{global}\left(W\right). \end{array}$$

**Figure 7:**
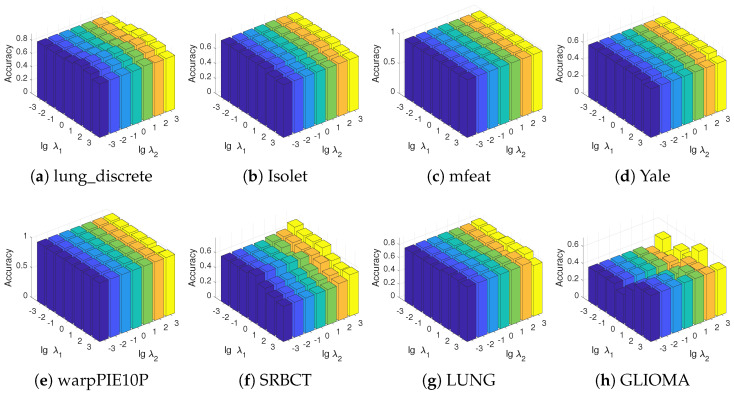
Parameter sensitivity study of the class-specific and global regularizations across all datasets. The parameters $\lambda_{1}$ and $\lambda_{2}$ correspond to the class-specific and global regularizations, respectively.

Figure 7 illustrates the influence of the parameters $\lambda_{1}$ and $\lambda_{2}$ on DRFS performance, where we set α=1 and the number of selected features to 50 for consistency. The results indicate that DRFS exhibits a general insensitivity to the variations in these parameters across most datasets, except for the SRBCT and GLIOMA datasets. Specifically, a distinct pattern emerges on the SRBCT dataset, where the model’s performance improves as $\lambda_{1}$ decreases and $\lambda_{2}$ increases. In contrast, on the GLIOMA dataset, larger values of $\lambda_{1}$ yield better performance. These observations suggest that the nature of intra-class feature associations and inter-class feature redundancy varies across different datasets. Since DRFS exhibits low sensitivity to $\lambda_{1}$ and $\lambda_{2}$ on most datasets, we recommend using the strategy in Equation (10) for efficient parameter adjustment.

**Figure 8:**
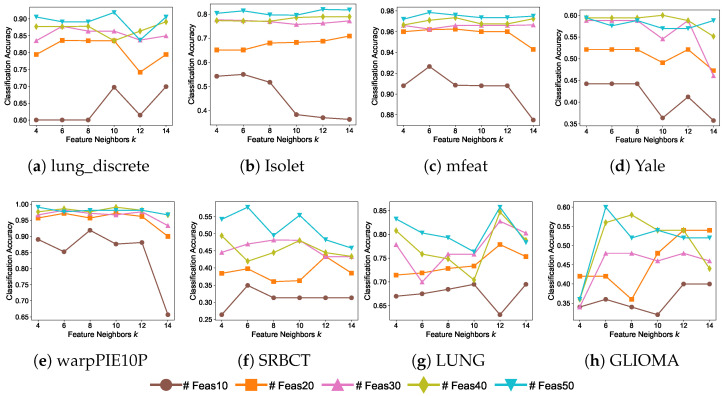
Accuracy of DRFS with varying feature neighbors *k*. “# Feas” denotes the number of selected features.

Figure 8 illustrates the impact of the number of feature neighbors, *k*, on classification performance across all datasets. The parameter *k* controls the neighborhood size used to calculate feature similarities, playing a critical role in representing feature associations. As shown in Figure 8, the effect of *k* on classification accuracy is closely related to the number of selected features. Specifically, the impact of *k* is more pronounced when fewer features are selected. For example, on the Isolet, mfeat, Yale, and warpPIE10P datasets, when only 10 features are selected, accuracy decreases as *k* increases. This suggests that with smaller feature sets, a larger neighborhood may introduce more feature associations affecting model, which can degrade model performance. Moreover, we observe that the SRBCT and GLIOMA datasets exhibit higher sensitivity to variations in *k*. This may be attributed to the inherent complexity of these datasets, which could lead to more intricate feature interactions. As evidenced by the parameter sensitivity analysis in Figure 6 and Figure 7, these datasets demonstrate more noticeable variations in performance. In this study, *k* is uniformly set to 10 to ensure consistency in capturing feature associations across all datasets.

### 5.6. Convergence Study

**Figure 9:**
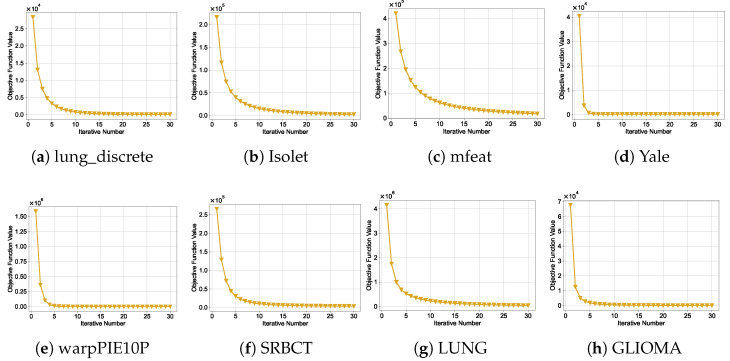
Convergence curves of DRFS across all datasets.

In Section 4.3, we provided a theoretical analysis of the convergence of Algorithm 1. In this subsection, we further investigate the convergence behavior of the objective function in (12) across iterations. Understanding the convergence of the DRFS algorithm is crucial to ensure its efficiency and stability when optimizing the feature selection process. Figure 9 shows the convergence curve of DRFS for various datasets. As shown, DRFS exhibits consistent and steady convergence, with the objective function value decreasing progressively until stabilization. In most cases, the algorithm reaches a stable solution within 30 iterations, highlighting its computational efficiency.

## 6. Conclusions

In this paper, we addressed the limitations of existing feature selection methods focusing solely on global feature associations. We highlighted the importance of incorporating class-specific feature interactions, which are essential for capturing patterns unique to individual classes. By proposing the Dual-Regularized Feature Selection (DRFS) method, we introduced a novel approach that simultaneously considers both class-specific and global feature associations. Our dual-regularization strategy ensures that important class-specific features are preserved while minimizing redundant global features. Importantly, our regularization technique is designed to function as an adaptable plugin, compatible with various feature selection models. The experimental results demonstrated that DRFS outperforms existing methods in classification tasks, offering a more effective and robust solution for feature selection.

However, our method has some limitations. Due to the fine-grained consideration of feature interactions, DRFS faces challenges in terms of storage and computation when dealing with very high dimensional features. Additionally, since our model is a shallow model, it may struggle to capture highly complex, non-linear relationships between features. In future work, we plan to address these challenges by optimizing the algorithm for high-dimensional data and exploring its integration with deep learning models to better capture non-linear interactions.

## Figures and Tables

**Table 1:** Summary of key feature similarity-based FS methods. For ‘Task’, ‘S’ for supervised, ‘SE’ for semi-supervised, and ‘U’ for unsupervised.

Method	Global Association	Class-Specific Association	Similarity Measure	Task
UnLasso [15]	√	×	Square cosine similarity	S
GRMOR [16]	√	×	Square cosine similarity	S
InteractedLasso [27]	√	×	Hypergraph	S
InElasticNet [28]	√	×	Information theory	S
InFusedLasso [29]	√	×	Information theory	S
SFSRM_MI [26]	√	×	Information theory	SE
SFSRM_P [26]	√	×	Pearson correlation coefficient	SE
AGLRM [30]	√	×	Gaussian function	SE
NSSRD [17]	√	×	Gaussian function	U
NSSRD_PF [17]	√	×	Parameter-free methods	U
NNSAFS [18]	√	×	Parameter-free methods	U
ManiFeSt [12]	×	√	Gaussian function	S
Inf-FS [31]	√	×	Weighted strategy	S/U
DRFS (ours)	√	√	Gaussian function	S

**Table 2:** Dataset description.

Dataset	# Features	# Samples	# Classes	Class Distribution	Type
lung_discrete	325	73	7	[6, 5, 5, 16, 7, 13, 21]	Bioinformatics
Isolet	617	1560	26	60 samples per class	Spoken Letter
mfeat	649	2000	10	200 samples per class	Image
Yale	1024	165	15	11 samples per class	Image
warpPIE10P	2420	210	10	21 samples per class	Image
SRBCT	2308	83	4	[29, 11, 18, 25]	Bioinformatics
LUNG	3312	203	5	[139, 17, 21, 20, 6]	Bioinformatics
GLIOMA	4434	50	4	[14, 7, 14, 15]	Bioinformatics

**Table 3:** Comparison of optimal classification accuracy across eight datasets using the SVM classifier. The best-performing method for each dataset is highlighted in **bold**, while the second-best result is underlined.

Dataset	AllFea	Fisher [5]	Gini Index [21]	RelieF [4]	RJFWL [24]	GRMOR [16]	Inf-FS [31]	ManiFeSt [12]	DRFS (ours)
lung_discrete	0.8514	0.9333	0.7943	0.8771	0.8924	0.8914	0.8924	0.8495	**0.9448**
Isolet	0.9635	0.8385	0.7660	0.7910	0.8622	0.8994	0.9141	0.7955	**0.9179**
mfeat	0.9870	0.9875	0.9635	0.9790	0.9850	0.9820	0.9760	0.9480	**0.9870**
Yale	0.7515	0.7636	0.5697	0.6848	0.6606	0.7152	0.6545	0.4242	**0.7697**
warpPIE10P	1.0000	0.9762	0.9429	0.9667	0.9952	**1.0000**	0.9810	**1.0000**	**1.0000**
SRBCT	1.0000	**1.0000**	**1.0000**	0.9647	**1.0000**	0.9647	**1.0000**	0.7728	**1.0000**
LUNG	0.9659	0.9510	0.9360	**0.9705**	0.9461	0.9657	0.9607	0.8572	0.9607
GLIOMA	0.7600	0.8200	0.8800	0.8200	0.8600	0.8800	0.8000	0.6400	**0.9200**

**Table 4:** Comparison of optimal classification accuracy across eight datasets using the 1-NN classifier. The best-performing method of each dataset is highlighted in **bold**, while the second-best result is underlined.

Dataset	AllFea	Fisher [5]	Gini Index [21]	RelieF [4]	RJFWL [24]	GRMOR [16]	Inf-FS [31]	ManiFeSt [12]	DRFS (ours)
lung_discrete	0.8362	0.8895	0.7676	0.8781	0.8371	0.8495	0.8505	0.8086	**0.9171**
Isolet	0.8686	0.8038	0.6667	0.6737	0.7583	0.8167	0.8314	0.6353	**0.8718**
mfeat	0.9810	0.9845	0.9315	0.9590	0.9775	0.9795	0.9785	0.9125	**0.9855**
Yale	0.6606	**0.6909**	0.5697	0.5758	0.6000	0.6788	0.6121	0.3515	0.6545
warpPIE10P	0.9571	0.9762	0.8571	0.9667	0.9905	0.9762	0.9714	0.9429	**1.0000**
SRBCT	0.8684	**1.0000**	0.9765	0.8926	**1.0000**	0.9294	0.9404	0.6397	**1.0000**
LUNG	0.9510	0.9460	0.9016	0.9312	0.9313	0.9557	**0.9609**	0.8230	0.9559
GLIOMA	0.7800	0.8000	0.8400	0.8000	0.8200	0.8400	0.8200	0.5000	**0.8600**

**Table 5:** Friedman test statistics $F_{F}$ and corresponding *p*-value for each metric. The critical value is 14.07 at a significance level of α=0.05.

Metric	$F_{F}$	*p*-Value
SVM	25.57	$6.02\times 10^{-4}$
1-NN	37.49	$3.78\times 10^{-6}$

**Table 6:** Ablation study showing average SVM classification performance with selected features in the range [10, 20, ⋯, 100].

Classifier	Regularization	Modules			
	Class-Specific Global	×	×	√	√
		×	√	×	√
SVM	lung_discrete	0.8380	0.9115	0.9171	**0.9226**
	Isolet	0.7683	0.8310	0.8318	**0.8320**
	mfeat	0.9729	0.9752	**0.9766**	**0.9766**
	Yale	0.5794	0.6982	0.7079	**0.7091**
	warpPIE10P	0.9790	0.9814	0.9871	**0.9881**
	SRBCT	0.9953	0.9965	0.9976	**1.0000**
	LUNG	0.9248	0.9257	0.9331	**0.9346**
	GLIOMA	0.8240	0.8460	0.8620	**0.8740**

**Table 7:** Ablation study showing average 1-NN classification performance with selected features in the range [10, 20, ⋯, 100].

Classifier	Regularization	Modules			
	Class-Specific Global	×	×	√	√
		×	√	×	√
1-NN	lung_discrete	0.8020	0.8770	0.8895	**0.8937**
	Isolet	0.6886	0.7744	0.7785	**0.7790**
	mfeat	0.9666	0.9758	**0.9780**	**0.9780**
	Yale	0.5103	0.6091	0.6109	**0.6164**
	warpPIE10P	0.9710	0.9824	0.9829	**0.9838**
	SRBCT	0.9929	0.9988	0.9988	**1.0000**
	LUNG	0.9007	0.8913	0.9292	**0.9307**
	GLIOMA	0.7860	0.7800	0.8320	**0.8500**

## Data Availability

The original data presented in the study are openly available in scikit-feature selection repository (https://jundongl.github.io/scikit-feature/datasets.html, accessed on 13 January 2025) or UCI machine learning repository (https://archive.ics.uci.edu/ml/datasets, accessed on 13 January 2025).
